# Magnetoelastic Effect-Based Transmissive Stress Detection for Steel Strips: Theory and Experiment

**DOI:** 10.3390/s16091382

**Published:** 2016-08-29

**Authors:** Qingdong Zhang, Yuanxiao Su, Liyuan Zhang, Jia Bi, Jiang Luo

**Affiliations:** School of Mechanical Engineering, University of Science and Technology Beijing, Beijing 100083, China; zhang_qd@me.ustb.edu.cn (Q.Z.); suyuanxiao@sina.com (Y.S.); bijia@ustb.edu.cn (J.B.); jixieluojiang@163.com (J.L.)

**Keywords:** magnetoelastic effect, stress detection, mathematical model, temperature compensation, magnetic shielding

## Abstract

For the deficiencies of traditional stress detection methods for steel strips in industrial production, this paper proposes a non-contact stress detection scheme based on the magnetoelastic effect. The theoretical model of the transmission-type stress detection is established, in which the output voltage and the tested stress obey a linear relation. Then, a stress detection device is built for the experiment, and Q235 steel under uniaxial tension is tested as an example. The result shows that the output voltage rises linearly with the increase of the tensile stress, consistent with the theoretical prediction. To ensure the accuracy of the stress detection method in actual application, the temperature compensation, magnetic shielding and some other key technologies are investigated to reduce the interference of the external factors, such as environment temperature and surrounding magnetic field. The present research develops the theoretical and experimental foundations for the magnetic stress detection system, which can be used for online non-contact monitoring of strip flatness-related stress (tension distribution or longitudinal residual stress) in the steel strip rolling process, the quality evaluation of strip flatness after rolling, the life and safety assessment of metal construction and other industrial production links.

## 1. Introduction

As a main product in the iron and steel industry, cold rolling steels have been an important material in all aspects of the national economy. With the improvement of the cold rolling technology, a higher and higher standard is specified for the shape quality of steel strips [1]. In the rolling production process, applying tension is a key means to control the steel shape and to guarantee a continuous rolling line, while a large tension would cover the possible shape defects of the steel strips. It is easy to cause stable fluctuation of the strips in their overall lengths, bringing difficulty to controlling the shape defects quantitatively [2]. After uncoiling or annealing treatment, the latent shape defects will be released, and a variety of flatness defects, for instance edge wave, central wave and composite wave, would appear [3]. A crucial factor to improve the shape quality of the cold rolling steel strips is to detect the stress distribution accurately [4].

Various stress detection devices have been developed, and some of them are used in industry. A popular form is the contact pressure-based detection device involving several separation rollers, by building the relation between the tension in the steel strip and the pressure on the rollers. However, this kind of device has a low sensitivity due to the contact disturbance of the strip and rollers. The contact measurement method may also scratch the strip and mold when slipping occurs. Besides, this tooling investigation is huge, and the performance cost ratio is relatively low [5,6]. The magnetic effect-based stress detection technique is a non-contact detection method that can conquer the deficiency of the traditional contact pressure detection method and, thus, becomes a new research hotspot [7,8,9,10,11,12,13].

Vourna et al. [7] and Kypris et al. [8] proposed the stress detection methods based on magnetic Barkhausen noise analysis and established the corresponding representation approaches. They employed an X-ray diffraction stress analysis method to verify the feasibility of their detection methods. Roskosz et al. [9] studied the residual magnetic field of a ferromagnetic material in the magnetization process and evaluated the residual stress by using the gradient of the residual magnetic field. Based on the geomagnetic field, Hu et al. [10] proposed a non-destructive testing method for thin-plate aluminum alloys. Yamada et al. [11], Sablik et al. [12] and Liu et al. [13] proposed the magnetic anisotropy-based stress detection method using the quadripolar sensor. Liu et al. [13] also derived the explicit relation between the detected voltage and the measured stress. The sensor in [11,12,13] was placed on the same side of the sample, which limits the selection of the magnetic core. Furthermore, the gap between the sensor and the sample is not easy to control in practice. The above methods are mainly utilized to detect the structural concentrated stress and welding residual stress of some ferromagnetic materials. However, little research is reported for magnetoelastic effect-based transmissive stress detection of the steel strips.

Our group has done some experiments on the magnetoelastic effect-based stress detection method, in which the locations and parameters of the sensors and the distance between the steel strip and sensors are studied [14,15]. This paper presents further systematic investigations on the theoretical model and the compensation for the measurement errors caused by the external environment factors (e.g., temperature and magnetic field). The mathematical model of the magnetoelastic effect-based transmissive stress detection will be established to obtain the explicit relation between the output voltage and the loaded stress. The stress measurement device will be designed to validate the theoretical model and to conduct repeatability verification tests. In addition, the influences of some external factors on the detection accuracy of the device will be investigated, and the correction schemes will be proposed correspondingly.

## 2. Basic Principle of Electromagnetic Detection

When the ferromagnetic material is magnetized by an external magnetic field, the length or the volume of the ferromagnetic material will change due to the deformation of the crystals. This phenomenon is called the magnetostrictive effect, including linear magnetostriction and volume magnetostriction. The linear magnetostriction results from the changes of the anisotropy energy and the magnetoelastic energy in crystals. Thus, the magnetostriction in a monocrystal is anisotropic. The length change in the magnetostrictive effect was first observed by James Joule in 1842 (Joule effect). The volume magnetoelastic effect results from the effect of exchange energy, which is isotropous. The volume magnetoelastic effect generally changes slightly, and it becomes obvious in the paramagnetic magnetization process after the technical magnetization reaches saturation. Thus, it is the linear (Joule) magnetostriction effect dominant in the case of steels. It is only needed to consider the linear magnetostriction effect in the ferromagnetic materials whose magnetizing curves are complex.

When the ferromagnetic material is subject to deformation or under external stress, the state of the magnetization of the material will change accordingly. This phenomenon is called the magnetoelastic effect, first discovered by Villari in 1865 (Villari effect) [16]. In the original approach to the issue of the magnetoelastic effect, this process is assumed to be reversible. The length of the magnetic material changes when the material is magnetized. It is expected that its magnetization will change when the material is strained, which was discussed by Bozorth [17]. Later, Based on Le Chatelier’s principle, Cullity established the thermodynamics relation equation between stress and magnetic flux density. However, for ferromagnetic materials, the magnetization process is hysteretic and therefore inherently irreversible in nature, although reversible changes in magnetization are superposed on the irreversible changes. Therefore, Jiles established the theoretical model of the magnetomechanical effect for ferromagnetic materials under unidirectional stress. There are reversible magnetization and irreversible magnetization in the magnetization process. The magnetization intensity of ferromagnetic materials is related not only to the stress, but also to the anhysteretic remanent magnetization [18]. From the viewpoint of energy, when the ferromagnetic material is under stress, its crystal will become deformed accordingly. The energy in the crystals includes not only the anisotropy energy from the spontaneous deformation, but also the magnetoelastic energy from the deformation caused by the external stress.

When the material is in the scope of elastic deformation, the stress will affect the external shape of the material. For a steel strip under uniaxial tension, the longitudinal tension in the non-rolling area will cause the stress changes of the steel plate in the rolling direction. The steel plate is stretched in the rolling direction and compressed in the plate width direction. Under the role of the magnetoelastic effect, the magnetic domain in the ferromagnetic steel plate is deflected to the stress direction, causing the changes in the overall permeability of the material. In this case, the tensile deformation of the steel plate along the rolling direction and the compressive deformation along the plate width direction due to the stress will cause the magnetic anisotropy of the isotropous ferromagnetic steel plate, i.e., the magnetic anisotropic effect caused by stress. The permeability in each direction of the determined angle of the steel plate with the tension is subject to regular changes accordingly, resulting in the changes in the magnetic flux density in the steel plate.

If a known magnetic field *H* is applied on an external ferromagnetic steel plate, the overall or partial magnetization state of the steel plate will be determined by its stress state and other relevant factors. When the influence of other external conditions such as temperature and magnetic disturbance on the steel plate magnetization state is shielded, the magnetic susceptibility of the steel plate and its permeability *μ* in each direction will change only due to the influence of the stress state. After establishing a determined magneto-mechanical model [19] and designing a series of appropriate sensors and relevant circuits, the stress in the steel plate strip can be calculated according to the increment of the permeability ∆*µ*, and the residual stress causing the uneven distribution of the steel plate stress can also be obtained. The purpose of stress measurement is to convert the stress/strain signal (which is hard to measure directly) into a measurable electric signal [20].

## 3. Mathematical Model of Transmissive Stress Detection Based on the Magnetoelastic Effect

### 3.1. Analysis of a Magnetic Circuit

**Figure 1:**
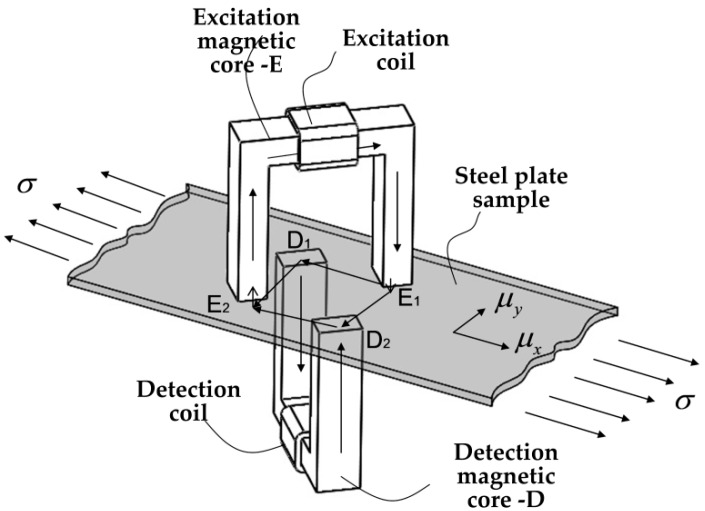
Schematic diagram of the magnetic penetration stress detection methods based on magnetic anisotropy.

As shown in Figure 1, a double-sided transmission-type quadripolar sensor stress measurement system is designed in this paper, according to the principle of ferromagnetic material anisotropy caused by the magnetoelastic effect in the stress state. When an alternating current is applied in the excitation magnetic coil, an alternating magnetic field will be generated in the quadripolar sensor, and the magnetic flux will flow through excitation magnetic core. For simplicity, it is specified in the following description that the fluxes outside the excitation magnetic core are from *E*_1_ to *E*_2_. There are many magnetic fluxes between *E*_1_ and *E*_2_. Most of them directly run from *E*_1_ to *E*_2_ along the steel plate via the shortest straight path, while the rest along other magnetic paths include the magnetic induction lines *E*_1_→*D*_1_→*E*_2_ and *E*_1_→*D*_2_→*E*_2_.

If the tested piece is made of isotropic ferromagnetic materials free of external force, the magnetic field intensity at *D*_1_ and *D*_2_ is the same. Therefore, the flux through the detection core is zero, and the induced electromotive force is not generated in the detection coil. In this case, the output voltage of the sensor is zero in theory. When the tested piece is under an external load, however, the material permeabilities along different directions will change accordingly, resulting in the magnetic anisotropy of the material. In this case, the magnetic field intensities at *D*_1_ and *D*_2_ will be different, producing different magnetomotive forces. Further, when the detection magnetic circuit is located between *D*_1_ and *D*_2_, the magnetic flux will be generated in the detection core. According to Faraday’s law of electromagnetic induction, the electric current will also be generated in the detection coil. The variation of the voltage signal with respect to the stress can be obtained.

### 3.2. Relation Formula between Output Voltage and the Loaded Stress

**Figure 2:**
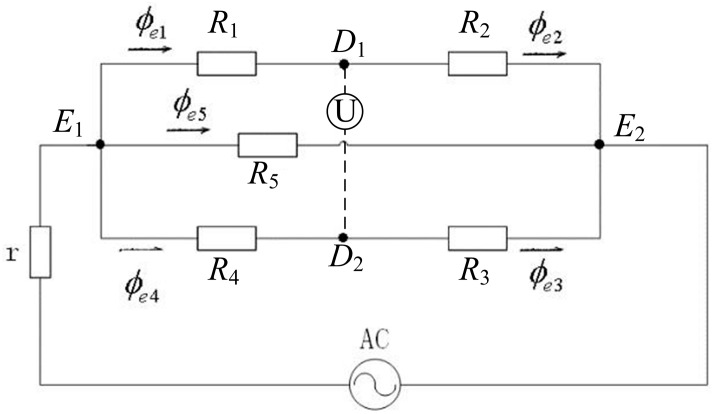
Schematic diagram of the sensor’s excitation magnetic equivalent circuit and magnetic flow.

To obtain the relation between the output voltage by the detection sensor and the stress, it is required to establish the recurrence relation from the stress to permeability change and then to the magnetic flux change. Here, it is assumed that the introduction of the detection core would not change the flux flows in the excitation magnetic circuit [11,13]. This is a mechanical-electric-magnetic coupling model of the magnetic detection voltage signal. To this end, the excitation circuit and magnetic detection circuit need be analyzed, as shown in Figure 1. Here, the reluctances of the detected steel plate between different probes of the sensor are denoted as $R_{1},R_{2},R_{3},R_{4}$ and $R_{5}$, respectively. The equivalent magnetic circuits of the excitation magnetic circuits are illustrated in Figure 2, where $\phi_{e1},\phi_{e2},\phi_{e3},\phi_{e4},\phi_{e5}$ are the fluxes through the reluctance $R_{1},R_{2},R_{3},R_{4},R_{5}$. It should be noticed that the voltmeter in Figure 2 is a schematic to illustrate the detection principle intuitively, and it does not belong to the excitation magnetic circuit.

**Figure 3:**
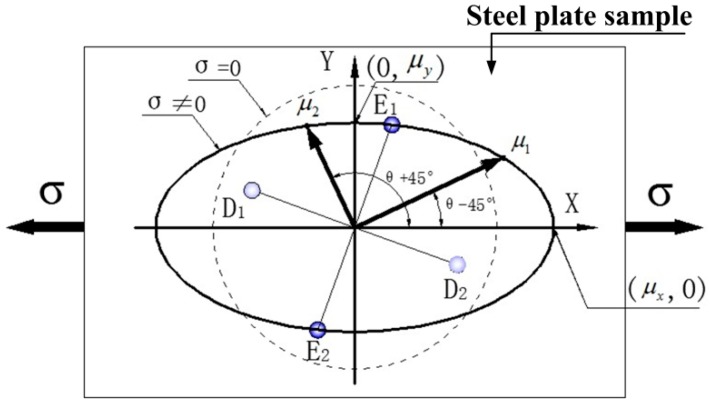
Elliptical distribution diagram of permeability in a steel plate under the stress state.

The magnetic anisotropy is generated when the original isotropous ferromagnetic steel plate is subject to residual stress or external force. At this time, the permeability along each direction in the steel plate presents an elliptical distribution law [11]. When the steel plate is under uniaxial tension, the permeability distribution is shown in Figure 3. Compared to the permeability distribution without stress (dotted circle), the permeability parallel to the loading force increases with the force, while the permeability perpendicular to the force decreases due to Poisson’s ratio. In the case shown in Figure 3, the magnetic circuit direction of the reluctance $R_{1}$ and $R_{3}$ is parallel; the changes of the permeability $\mu_{1}$ and $\mu_{3}$ along directions *E*_1_*-D*_1_ and *E*_2_*-D*_2_ are the same, so the corresponding reluctances $R_{1}$ and $R_{3}$ always keep equal. Meanwhile, the permeability $\mu_{4}$ and $\mu_{2}$ and the reluctances $R_{4}$ and $R_{2}$ along directions *E*_1_*-D*_1_ and *E*_2_*-D*_2_ also keep equal. Thus, one has $R_{1}+R_{2}=R_{3}+R_{4}$.

At this time, the equivalent reluctance of the magnetic circuit is:

(1) $$R_{m}={\left(\frac{1}{R_{1}+R_{2}}+\frac{1}{R_{3}+R_{4}}+\frac{1}{R_{5}}\right)}^{-1}=\frac{(R_{1}+R_{4})R_{5}}{R_{1}+R_{4}+2R_{5}}$$

The total reluctance of the magnetic circuit can be expressed as:

(2) $$R_{me}=r+R_{e}+R_{m}$$

where r is the total air reluctance between the magnetic core and the detected steel plate and $R_{e}$ is the reluctance of the excitation magnetic core.

Because $R_{1}$ and $R_{2}$ are in a branch and $R_{3}$ and $R_{4}$ are in the other, it gets $\phi_{e1}=\phi_{e2}$ and $\phi_{e3}=\phi_{e4}$. Further, $R_{1}+R_{2}=R_{3}+R_{4}$ produces $\phi_{e1}=\phi_{e2}=\phi_{e3}=\phi_{e4}$. The total flux in the magnetic circuit is:

(3) $$\phi_{me}=2\phi_{e1}+\phi_{e5}$$

The magnetic flux is inversely proportional to reluctance in the parallel circuit, giving:

(4) $$\frac{\phi_{e1}}{\phi_{e5}}=\frac{R_{5}}{R_{1}+R_{4}}$$

Substituting it into the expression of $\phi_{me}$ produces:

(5) $$\phi_{me}=2\phi_{e1}+\frac{R_{1}+R_{4}}{R_{5}}\phi_{e1}=\frac{2R_{5}+R_{1}+R_{4}}{R_{5}}\phi_{e1}$$

Combining Equation (5) and $\phi_{me}=\frac{F_{me}}{R_{me}}=\frac{N_{e}}{R_{me}}i_{e}$, one has:

(6) $$\phi_{e1}=\frac{N_{e}R_{5}}{(r+R_{e})(R_{1}+R_{4}+2R_{5})+(R_{1}+R_{4})R_{5}}i_{e}$$

where $F_{me}$ is the total magnetomotive force of the magnetic circuit, $N_{e}$ is the number of turns of the excitation coil and $i_{e}$ is the excitation current.

To obtain the magnetic flux of the induction coil, it is required to obtain the difference of the magnetomotive force between *D*_1_ and *D*_2_ of the induction magnetic core, that is:

(7) $$F_{md}=F_{D1}-F_{D2}$$

Let the magnetomotive force in *E*_1_ be $f_{E1}$ and the magnetic pressure drops in $R_{1}$ and $R_{4}$ be $U_{R1}$ and $U_{R4}$, respectively. One has:

(8) $$F_{D1}=f_{E1}-U_{R1}$$

(9) $$F_{D2}=f_{E1}-U_{R4}$$

Substituting Equations (8) and (9) into (7) provides:

(10) $$F_{md}=U_{R4}-U_{R1}$$

Combined with Equation (6), when the steel plate is subject to residual stress or external force, the magnetic pressure drops in the reluctance $R_{1}$ and $R_{4}$ become:

(11) $$U_{R1}=\phi_{e1}R_{1}=\frac{N_{e}R_{5}R_{1}}{(r+R_{e})(R_{1}+R_{4}+2R_{5})+(R_{1}+R_{4})R_{5}}i_{e}$$

(12) $$U_{R4}=\phi_{e4}R_{4}=\frac{N_{e}R_{5}R_{4}}{(r+R_{e})(R_{1}+R_{4}+2R_{5})+(R_{1}+R_{4})R_{5}}i_{e}$$

Thus, the magnetomotive force difference between *D*_1_ and *D*_2_ can be expressed as:

(13) $$F_{md}=U_{R4}-U_{R1}=\frac{N_{e}R_{5}(R_{4}-R_{1})}{(r+R_{e})(R_{1}+R_{4}+2R_{5})+(R_{1}+R_{4})R_{5}}i_{e}$$

The magnetomotive force difference will generate alternating flux in the detecting poles, i.e.,

(14) $$\Phi_{i}=\frac{F_{md}}{r_{2}+R_{d}}$$

where $r_{2}$ is the total air reluctance between the detecting pole and the detected steel plate and $R_{d}$ is the reluctance of the detecting pole core.

Therefore, the magnetic flux through the detecting pole is:

(15) $$\Phi_{i}=\frac{N_{e}R_{5}(R_{4}-R_{1})}{\left[(r+R_{e})(R_{1}+R_{4}+2R_{5})+(R_{1}+R_{4})R_{5}\right](r_{2}+R_{d})}i_{e}$$

To simplify the expression, let $r_{1}$ denote the total air reluctance between the excitation magnetic pole and the detected steel plate and $r_{2}$ denote the total air reluctance between the detecting pole and the detected steel plate. Since the air permeability is far less than the measured steel plate and sensor magnetic core permeability, it is available: $R_{1},R_{4},R_{5},R_{e},R_{d}<<r_{1},r_{2},r$. Thus, the above equation can be approximated to:

(16) $$\Phi_{i}\approx \frac{N_{e}R_{5}(R_{4}-R_{1})}{\left(r_{1}+r_{2})r_{2}(R_{1}+R_{4}+2R_{5}\right)}i_{e}$$

According to the theory of electromagnetism, the total flux through the detection coil with the number of turns of $N_{d}$ is:

(17) $$\Psi_{d}=N_{d}\Phi_{d}$$

It can be known from Faraday’s law of electromagnetic induction that the induced electromotive force generated in the measurement coil is:

(18) $$\varepsilon =-\frac{d\Psi_{d}}{dt}$$

When the stress in the detected piece keeps unchanged, the induced electromotive force in the measurement coil becomes:

(19) $$\varepsilon =\frac{-N_{e}N_{d}}{r_{2}(r_{1}+r_{2})}\frac{R_{5}(R_{4}-R_{1})}{R_{1}+R_{4}+2R_{5}}\frac{di_{e}}{dt}$$

Due to $R_{1},R_{4},R_{5},<<r_{1},r_{2}$ in the above formula, one has:

(20) $$\varepsilon \propto k_{1}N_{e}N_{d}(R_{4}-R_{1})\frac{di_{e}}{dt}$$

Let the excitation current be subject to regular changes according to sine $i_{e}=I_{e}\sin(\omega t+\varphi )$. This case gives:

(21) $$\varepsilon \propto k_{1}\omega I_{e}N_{e}N_{d}(R_{4}-R_{1})\cos(\omega t+\varphi )$$

The effective value of the electromotive force generated in the detection coil is:

(22) $$\varepsilon \propto \frac{1}{2\sqrt{2}}k_{1}\omega I_{e}N_{e}N_{d}(R_{4}-R_{1})$$

According to the relation $R=\frac{l}{\mu S}$ between the reluctance and the permeability, the effective value of the output voltage in the corresponding detection coil is:

(23) $$V_{d}=\frac{\varepsilon }{\sqrt{R_{i}^{2}+X_{i}^{2}}}\propto k_{2}N_{d}N_{e}I_{e}(\frac{1}{\mu_{4}}-\frac{1}{\mu_{1}})$$

where l is the length of the magnetic circuit, S is the sectional area of the magnetic circuit, $R_{i}$ is the resistance value of the measurement coil and $X_{i}$ is the inductive reactance of the measurement coil.

According to the elliptical distribution diagram of the permeability in the stress state shown in Figure 3, it can be obtained that:

(24) $$\mu_{1}=\frac{\mu_{y}}{\sqrt{1-\left(1-\frac{{\mu_{y}}^{2}}{{\mu_{x}}^{2}}\right)\cos^{2}(\theta -45^{\circ })}}$$

(25) $$\mu_{4}=\frac{\mu_{y}}{\sqrt{1-\left(1-\frac{{\mu_{y}}^{2}}{{\mu_{x}}^{2}}\right)\sin^{2}(\theta -45^{\circ })}}$$

where $\mu_{x}$ and $\mu_{y}$ denote the relative permeability in the directions of *x* and *y*, respectively. Here, *x* is along the easy magnetization direction, and *y* is along the hard magnetization direction.

Substituting the above formula into Equation (22) with the condition $\theta =45^{\circ }$, the maximum value of the output voltage is:

(26) $$V_{mag}\propto k_{3}N_{d}N_{e}I_{e}(\frac{1}{\mu_{y}}-\frac{1}{\mu_{x}})=k_{3}N_{d}N_{e}I_{e}\frac{1}{\mu_{x}\mu_{y}}(\mu_{x}-\mu_{y})$$

At the reversible magnetization stage within the elastic deformation range of the material, its relationship with the stress is assumed as follows:

(27) $$\mu_{x}=\zeta \sigma +\mu_{e}$$

(28) $$\mu_{y}=-\frac{\zeta }{m}\sigma +\mu_{e}$$

where ζ is the correlation coefficient of the material and applied magnetic field, m is the ratio of the permeability increment along the easy magnetization direction under the role of stress to the permeability decrease along the hard magnetization direction and $\mu_{e}$ is initial permeability.

When the permeability increment Δμ caused by the stress is far less than the initial permeability $\mu_{e}$ of the material without stress, Equation (25) can be simplified as:

(29) $$V_{mag}\propto k_{4}N_{d}N_{e}I_{e}(\mu_{x}-\mu_{y})=kN_{d}N_{e}I_{e}\frac{\zeta (1+m)}{m}\sigma$$

It can be seen that when it is without regard to the magnetic flux leakage of the sensor and a part of the flux directly transmitted by the excitation coil to the induction coil bypasses the steel plate, the effective value of output voltage from the detection coil is directly proportional to the difference between the permeability increment along the easy magnetization direction in the detected piece and the permeability along the hard magnetization direction. When the material is at the reversible magnetization stage within the elastic deformation range of the material, the increment of the output voltage from the transmissive stress detection system is linearly related to the loaded stress.

## 4. Brief Introduction of the Transmissive Stress Detection Experimental Device

According to the actual production environment conditions on the cold-rolled steel strip site, a double-sided transmissive quadripolar sensor stress detection system is designed in this paper, with the sensor arrangement form, as shown in Figure 1. A pair of poles are placed orthogonally and placed without contact with the detected steel plate along the direction of 45° with the axis. Relative to the traditional one-sided sensor, the advantages of this arrangement form are mainly reflected in the following two aspects. Firstly, the excitation sensor and the detection sensor will not be subject to mechanical contact in the winding process of the sensor coil, so as to provide the possibility to select the sensors of more specifications and sizes. Secondly, the inevitable steel strip chattering phenomenon in the actual production process will cause changes in the direct clearance between the sensor and the steel plate and affect the detection results. The contralateral arrangement form achieves clearance compensation more easily [14].

**Figure 4:**
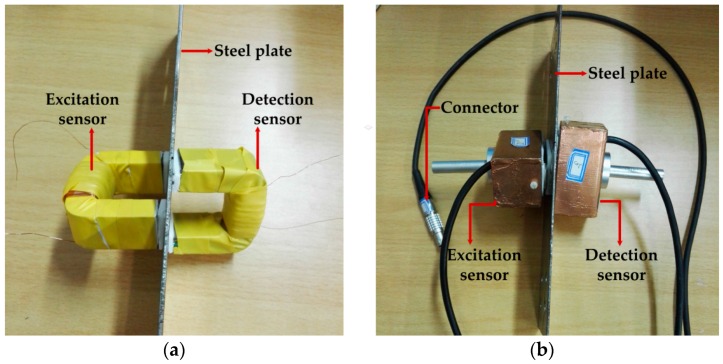
The installation diagram of the excitation sensor and detection sensor: (**a**) the sensors without magnetic shielding; (**b**) the sensors with the magnetic shielding and connector.

The sensor, located in the most significant end of the whole system, is used to pick up the variation of the induced electromagnetic field caused by the stress. Its detection sensitivity directly affects the whole performance. As an important medium to excite and detect the magnetic field generated by the system and to obtain signals, the sensor converts and detects the electrical signal and magnetic signal and directly affects the signal quality and detection results. To combine the sensor and the detected piece well into a magnetic loop, a common *U* magnetic core is selected. To ensure good magnetic conductive characteristics of the sensor magnetic core, the soft magnetic ferrite with the initial permeability of $\mu_{i}=2300\pm 25\%$ is selected as the probe magnetic core material. The excitation sensor and the detection sensor designed and made by us are shown in Figure 4.

**Figure 5:**
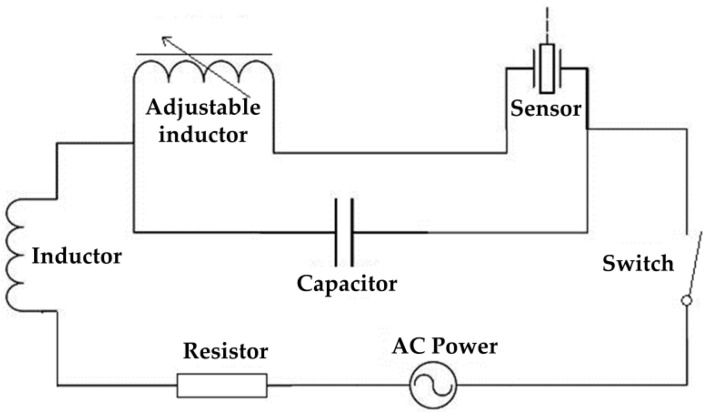
Schematic diagram of the excitation resonant circuit.

The electric power supply plan composed of the power frequency transformer and voltage regulator, with the operating circuit frequency of 50 Hz, is selected as the excitation source in the experiment designed. This design mode is achieved readily and can preliminarily meet the system requirements. The inductance in the experiment is connected by an adjustable inductance and fixed inductance in series. The capacitance is connected in parallel by a number of non-polarized capacitors [15]. Because AC power frequency in China is 50 Hz, this popular frequency is used in the experiment as the excitation source, and the inductance is adjusted to reach the resonance state. The excitation resonance circuit is shown in Figure 5.

**Figure 6:**
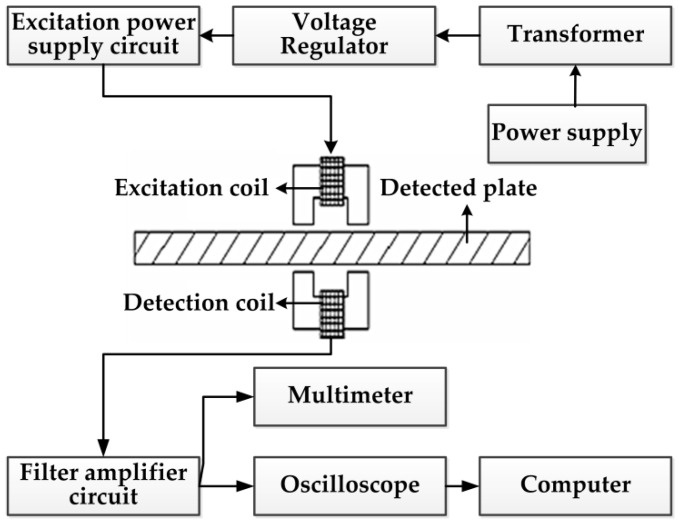
Principle diagram of the magnetic stress detection system.

**Figure 7:**
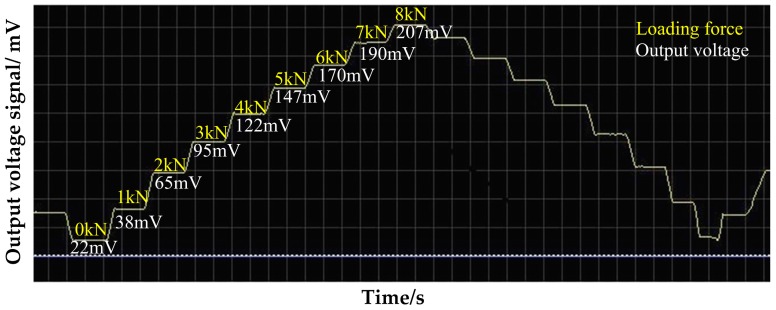
The output interface of the stress detection system.

According to the abovementioned principle, the magnetic measurement system of the stress is basically composed of the excitation system, sensor, signal detection system and signal processing system. The load is applied to the steel plate by setting an external stress loading system. The principle block diagram of the magnetic detection system is shown in Figure 6, in which the excitation system mainly provides the excitation current for the probes to generate a stable excitation field. Thus, the steel plate is in the reversible magnetization state, the sensor is classified into the excitation sensor and detection sensor and the signal detection system receives the voltage signal of the induced electromotive force changed with the applied load in the detecting sensor. Due to the relatively weak signal changes caused by applied stress in the actual stress detection process based on the magnetoelastic effect, it is required to use the filter and amplification technology to detect and output the weak signals. The signal processing system stores and processes the received signals to obtain the required data and displays in the oscillogram. The output interface displayed on the computer is shown in Figure 7.

## 5. The Experiment of External Factor’s Influences 

### 5.1. Repetitive Verification Experiment of the Detection System

**Figure 8:**
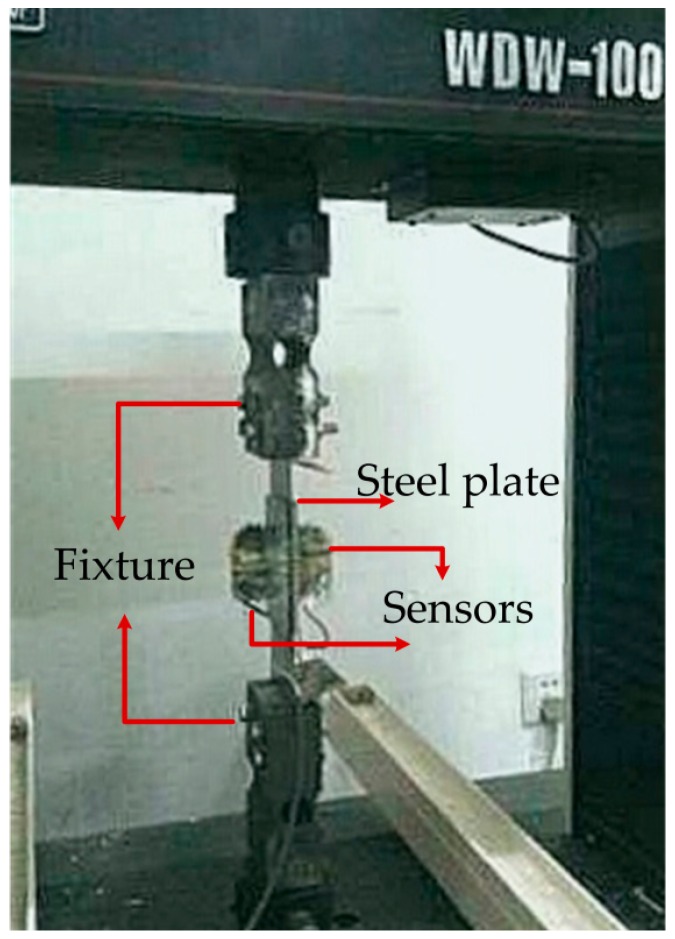
Photo of the magnetic stress detection system in working condition.

In this experiment, the above magnetoelastic stress detection method is subject to actual detection verification firstly. A set of systems that can automatically load the stress is set up under the experimental conditions. The whole system mainly consists of a WSW-10 microcomputer control electronic universal testing machine produced by Changchun Kexing, a hardware experiment platform and a computer, in which the testing machine can provide up to 100 kN tension, and its loading mode can be set through the PC program to achieve different loading processes. The steel plate detected piece, with the cross-section of 80 mm × 2 mm, is made of carbon structural steel Q235 in supply. Before the experiment, the residual stress of all sample steel plates was detected. The samples with low and similar residual stress were selected for the present study. The detected steel plate is accurately lineated before the sensor is fixed in order to ensure that the detection coil is 45° with the principal stress direction of the detected piece. The two sensors are perpendicular. In addition, the space between the U magnetic core and the steel plate is 0.3–0.4 mm; the excitation coil has 300 turns; and the induction coil has 600 turns. The clamping mode of the detection machine and the steel plate are shown in Figure 8.

**Figure 9:**
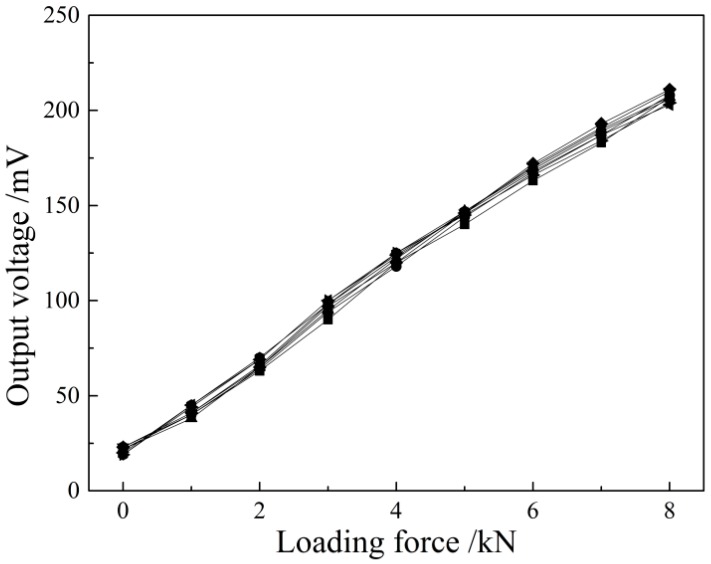
The output voltage with loaded stress of Q235 by repetitive experiment.

To verify the correctness of the mathematical model $V_{mag}\propto kN_{d}N_{e}I_{e}\frac{\zeta (1+m)}{m}\sigma$ of the above magnetic detection output signal and the stress, one of the key points is to observe whether the increment of the output voltage by the system is linearly related to the applied stress when the relevant parameters of the sensor and the detected piece of material are determined. The Q235 steel plate of 2 mm thickness is loaded eight times repeatedly at the excitation current of 150 mA. The loading process is by segments with the full-scale loading capacity of 8 kN and a loading step of 1 kN tension. The retention time of the load in each loading segment is 10–20 s. The experiment results in Figure 9 show that the detected voltage signal is linearly increased with the increase in the applied load in the loading process. If the experiment was truly started off with an isotropic sample without internal stress, the initial voltage signal would be 0 mV theoretically. The data curves will start from the coordinate (0,0), but the slope of the data curves will not be affected. In Figure 9, it is found that the voltage signal is about 21 mV in this experiment in the absence of applied load, because there is basically no steel plate with the internal stress of zero in actual life. The steel plate under the supply state is selected in this experiment and has certain residual stress before loading. Thus, it is reasonable and inevitable to detect a voltage signal before loading. Meanwhile, the external temperature and the external magnetic field may have certain influence on the voltage output signal, which will be specified in the following experimental study. According to the experimental results, the sensitivity detected under this parameter is 23.05 mV/kN, and the experimental results have ideal repeatability and can fully verify the mathematical model established above.

According to the experiment results, in order to get the relationship between the stress value in the steel plate and the output voltage, the experiment data need to be calibrated. From the formula $V_{mag}\propto kN_{d}N_{e}I_{e}\frac{\zeta (1+m)}{m}\sigma$, the coefficient k can be calculated. In the calibration experiment, the excitation coil has 300 turns; the induction coil has 600 turns; and the excitation current is 150 mA; the increment of the output voltage by the system is linearly related to the applied stress. Thus, the relation between the stress and the voltage can be given as $\sigma (\mathrm{MPa})=-5.6+0.2607V_{mag}(\text{mV})$, where the correlation coefficient is *R*^2^ = 0.9965.

**Figure 10:**
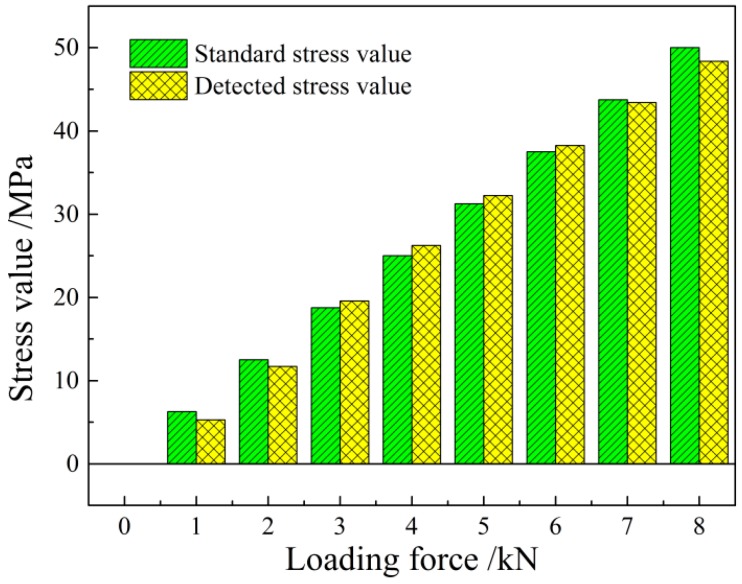
The comparison histogram of the standard and detected stress value.

Compare the standard stress value with the detected stress value in the calibration experiment; the comparison histogram is shown as Figure 10. It shows that in all of the loading force calibration experiments, the difference between the standard and detected stress is relatively small. According to the variance analysis of the experiment data from the repeated loading experiment, the repeatability error of the detection system is 2.15%, which indicates the high precision of the detection system, and it can meet the site measurement requirements. Besides, the present detection system in our experiment is the primary prototype. We believe that the sensitivity of the present method can be improved in further research.

### 5.2. Temperature Influence Analysis of the Detection System

The magnetization intensity of the ferromagnetic material with a definite type in the magnetic field is related to the external magnetic field intensity, external environment temperature and its own stress state [18,21]. To obtain the single correspondence of the material magnetization state with the applied load, it is required to eliminate the interference from the other two external factors. This section mainly studies the influence of the environment temperature. In an actual production environment, the steel strip temperature is related to the technological parameters in the site rolling production process, and it is difficult to control the temperature variation of the tested steel strip. The experiment for the influence on the sensor temperature characteristics and related temperature compensation technology become the key to test the effect of the method. The temperature influence curve of the magnetic measurement system is plotted through experiment as follows.

**Figure 11:**
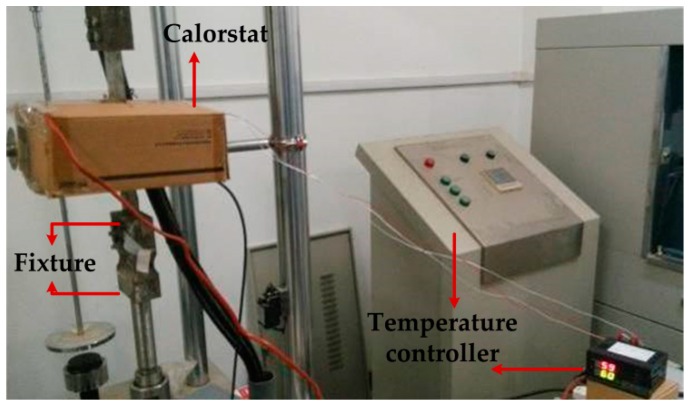
The stress testing experiment in the constant control of the temperature controller.

As shown in Figure 10, the sensors with magnetic shielding are placed in the calorstat, and the steel strip test piece is loaded by a universal tensile machine gradually from 0 to 8 kN with the loading step of 1 kN. Considering the actual situation of industrial application, the normal working temperature is generally in the range of 25–75 °C, and the room temperature is 27.9 °C. Therefore, the loading process is performed by four temperature nodes, i.e., 27.9 °C, 45 °C, 60 °C and 75 °C. Temperature is kept for 10 min at each test point, and then, the sensor is loaded from 0 to 8 kN to ensure an even temperature field. The sensor output is recorded, and the detection voltage under four temperatures can be detected to produce the curve shown in Figure 11.

**Figure 12:**
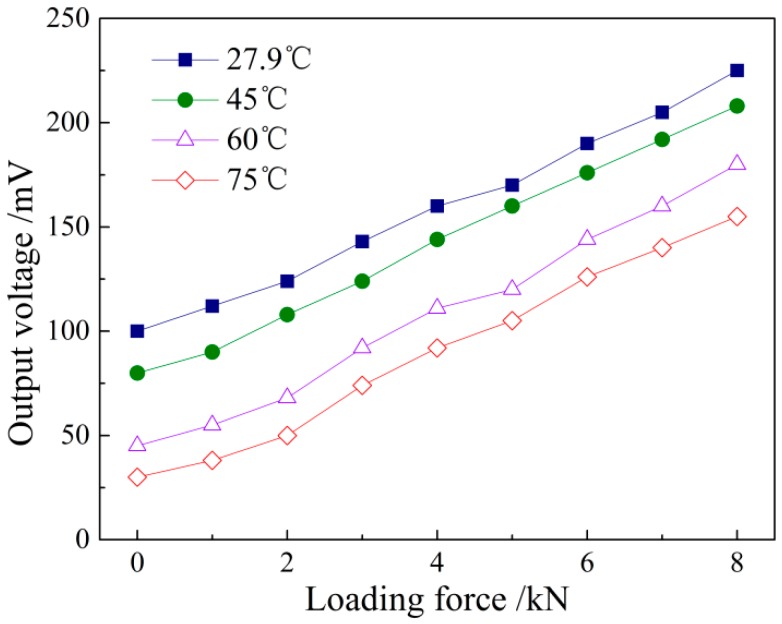
The output voltage with loaded stress at different temperatures.

The above figure shows that the output voltage detected by the sensor presents a declining trend on the whole with the loading force changes under the fixed loading force with the increase in the temperature. The temperature under the experiment conditions is less than the Curie temperature of Q235, and the experiment is in a low temperature state. According to the spin wave theory of Bloch [22], the magnetization intensity or permeability of ferromagnetic material is largely affected by the molecular field in the crystals. When the temperature is below the Curie point, the atomic thermal motion will be intensified with the temperature rise, and the thermal motion energy of the atomic magnetic moment is increased, resulting in the increase of the exchange energy. Therefore, the contribution of the molecular field to the magnetization intensity is weakened, the disorderly arrangement trend of the atomic magnetic moment is increased and the magnetization intensity *M* is decreased. In the temperature range studied in this paper, the permeability decreases as the temperature rises, affecting the effect of the stress action of the magnetoelastic effect in the test and generating the phenomenon shown in Figure 12. The output voltage signal shows a declining trend.

The temperature compensation of the linear sensor is mainly the compensation for zero position and sensitivity. The temperature compensation idea is to measure the steel plate temperature in the stress detection process of the sensor, and it performs the compensation by means of the temperature curve of the permeability to reduce or eliminate the influence of the temperature on the detection results. According to the detected data in the voltage signal output by the system, it is calculated that the relative change of the slope is 0.109% per degree, which can be ignored. Therefore, it is the zero position that is mainly affected by the temperature. Due to the temperature influence, the sensitivity is nearly unchanged. In the temperature rise of 47.1 °C, the total zero position falls by 70 mV. It can be assumed that the relationship between the initial voltage value $Y_{U}$ and the temperature is $Y_{U0}=a+b\Delta t$. Specifically, it is $Y_{U0}=100-1.486\times \Delta t$ to correct the initial value of the voltage signal.

### 5.3. Magnetic Shielding Influence Analysis of the Detection System

**Figure 13:**
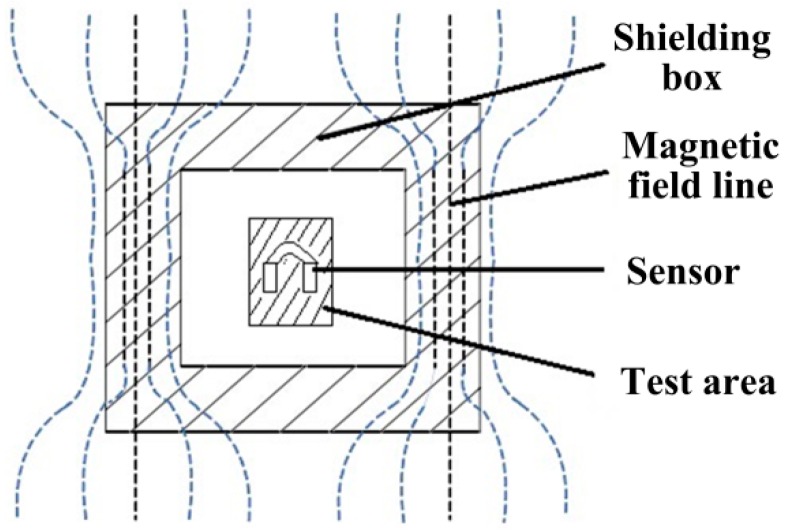
Principle diagram of the low frequency magnetic shield.

As mentioned above, to obtain the single correspondence of the material magnetization state with the loaded stress, it is required to eliminate the interference from external factors. This section mainly studies the influence of the external magnetic field on the detection effect, as well as the relevant shielding technology. In the real environment, various factors may shift the data. For example, the geomagnetic field, the machinery itself and other electronics in the laboratory. Furthermore, more factors will be encountered if the present detection method is applied in the industrial field. For the shielding of a low frequency magnetic field, the high permeability material may be used as the shield according to the shielding principle that the external disturbing magnetic field is shunted through the high permeability of the shield [23]. The shielding schematic diagram of the low frequency magnetic field is shown in Figure 13. In addition, the shielding principle of the high frequency magnetic field is using the eddy-current counter magnetic field generated on the surface of the shield by electromagnetic induction. Therefore, the conductivity of the material in the shielding box needs to be high to ensure the shielding effect.

The shield of a multilayer structure is designed, and the low-carbon steel box subject after annealing treatment is used as the shielding box with a certain intensity. The silicon steel sheet, which is a soft magnetic material with high magnetic permeability, is pasted internally. The sensor is placed in the shielding box, and the double-sided shielding copper tape with high conductivity is wound outside the magnetic shielding box to enhance the shielding effect. To shield the interference of the external environment to the signal transmission process, the twisted pair is used as the signal line of the sensor to reduce the capacitive and inductive coupling, improving the system common-mode rejection capability.

For comparative study of different output voltage signals detected by the system before and after shielding, the unshielded and shielded sensors are used to detect the same sample steel plate loading from 0 to 10 kN five times repeatedly. In this part, all of the experiments were conducted under the room temperature of 27.9 °C.

**Figure 14:**
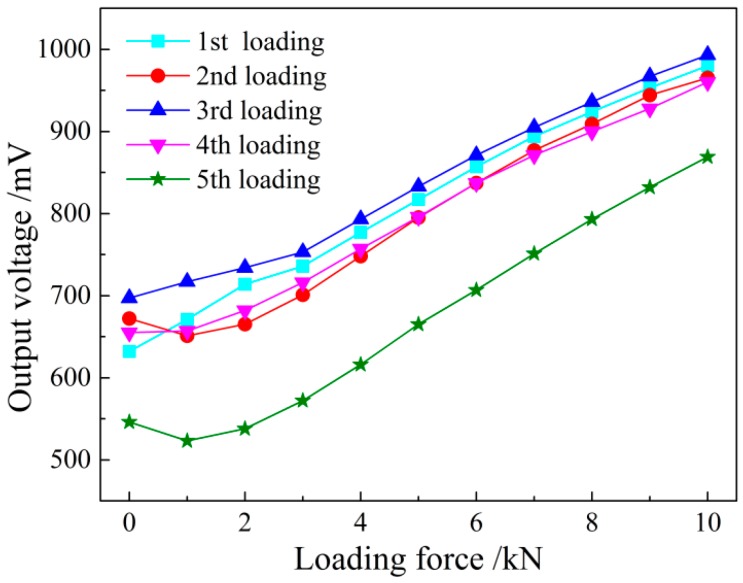
The output voltage with loaded stress without magnetic shielding.

**Figure 15:**
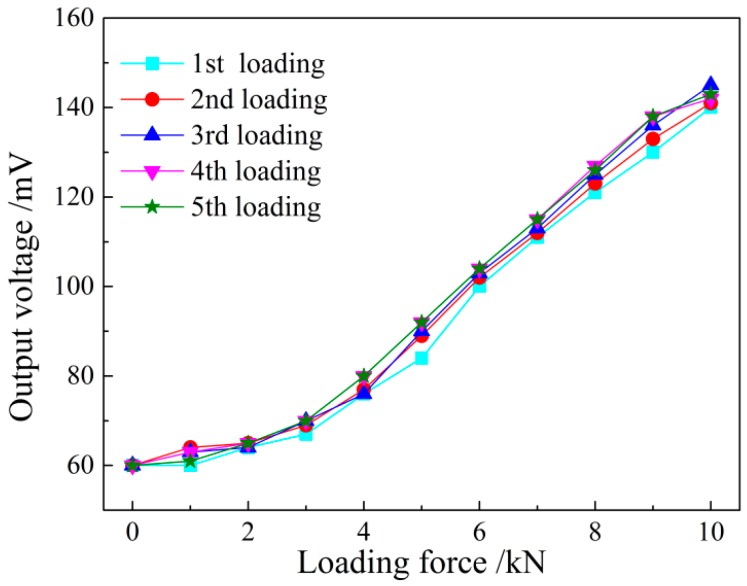
The output voltage with loaded stress with magnetic shielding.

The test process is shown in above segment, and the test results are shown in Figure 14 and Figure 15. It can be seen that the unshielded sensor is not of obvious repeatability due to the large interference from the external magnetic field, which is mainly reflected in the instable initial induced voltage value in a relatively high position, resulting in the disunity of the voltage curve with the loading force changes. The permeability of the magnetic shield is far higher than that of the air. Thus, most magnetic lines of force in the external environment pass through the air gap in the shielding box along its outer wall, and few magnetic lines of force reach the iron core of the sensor. The detected initial induced voltage value decreases obviously after shielding, and the five magnetic loading tests have good repeatability. The amplitude change of the voltage signal reduces, but the system output precision is greatly improved on the whole.

## 6. Conclusions

This paper proposes a magnetoelastic effect-based transmissive stress detection method and develops the sample device. The main conclusions are as follows:

1. Using Faraday’s law of the electromagnetic induction and equivalent magnetic circuit theory, this paper establishes the theoretical model of the magnetoelastic effect-based transmissive stress detection and obtains the explicit expression of the output voltage and the stress: $V_{mag}\propto kN_{d}N_{e}I_{e}\frac{\zeta (1+m)}{m}\sigma$. When the material is at the reversible magnetization stage within the elastic deformation range, the output voltage will be linearly related to its loaded stress.
2. The repeated experiment on Q235 steel under uniaxial tension shows that the detection voltage is linearly increased with the loaded stress, consistent with the theoretical prediction. The calibration experiment gives the stress-voltage relation $\sigma (\mathrm{MPa})=-5.6+0.2607V_{mag}(\mathrm{mV})$, with the correlation coefficient *R*^2^ = 0.9965. The repeatability error of the detection device is 2.15%, satisfying the industrial demands.
3. The external factors, including the environment temperature and surrounding magnetic field, disturb the stress detection. The temperature experiment shows that the detection voltage decreases with increasing the temperature in a certain range, while the detection sensitivity keeps unchanged. The temperature compensation formula $Y_{U0}=100-1.486\times \Delta t$ is proposed to correct the initial voltage value. The magnetic field distribution experiment shows that the sensor is largely interfered by the surrounding magnetic field. The initial voltage value is instable in a high position state, producing a disunity of the stress-voltage curve. Then, a magnetic shielding scheme is designed that improves the repeatability of the detection results effectively.

Our study, both theoretically and experimentally, verifies the feasibility of the transmissive stress detection method on the basis of the magnetoelastic effect. The present work establishes the foundations of the magnetic measurement stress system for various applications, which can be used for online non-contact stress detection of steel strips and other industrial links.
